# Impact of HIV diagnosis on job separation in Japanese employed men: a retrospective matched cohort study using an administrative claims database

**DOI:** 10.1186/s12889-026-27206-6

**Published:** 2026-04-15

**Authors:** Yuki Arisato, Kazuhiko Ikeuchi, Shinya Matsumoto, Toshiyuki Kishida, Akira Kado, Kazuya Okushin, Hiroshi Yotsuyanagi, Makoto Saito, Takeya Tsutsumi

**Affiliations:** 1 Department of Infectious Diseases, Graduate School of Medicine, The University of Tokyo, 7-3-1 Hongo, Bunkyo-ku, Tokyo, 113-8655 Japan; 2 Department of Infectious Diseases, Tokyo Metropolitan Cancer and Infectious Diseases Center Komagome Hospital, Tokyo, Japan; 3 Division for Health Service Promotion, The University of Tokyo, Tokyo, Japan; 4 Department of Infection Control and Prevention, Graduate School of Medicine, The University of Tokyo, Tokyo, Japan; 5 Department of Infectious Diseases and Applied Immunology, IMSUT Hospital of The Institute of Medical Science, The University of Tokyo, Tokyo, Japan; 6 Centre for Tropical Medicine and Global Health, Nuffield Department of Medicine, University of Oxford, Oxford, UK

**Keywords:** HIV, Job separation, Japan, Cohort study, Socioeconomic factors, Early diagnosis, Administrative claims database

## Abstract

**Background:**

People living with HIV (PLWH) have been reported to have higher unemployment rates than the general population. However, most previous studies were cross-sectional and lacked appropriate controls. To assess the impact of HIV diagnosis on job separation, we conducted a retrospective matched cohort study using a Japanese claims database of employed men.

**Methods:**

We conducted a retrospective matched cohort study of newly diagnosed male PLWH and non-PLWH controls using a Japanese administrative claims database mainly covering employees of larger enterprises. PLWH were matched 1:5 with controls based on age, dependent family status, psychiatric history, cancer history, and insurance enrollment month/year. The primary outcome was job separation within three years from diagnosis. For subgroup analyses, PLWH were stratified by prescriptions for *Pneumocystis jirovecii* pneumonia (PJP) prophylaxis or treatment as a proxy for CD4 count < 200 cells/µL.

**Results:**

A total of 1,031 newly diagnosed male PLWH and 5,155 matched controls were included. Overall, the PLWH group had a higher risk of job separation than the control group (three-year incidence: 36.8% vs. 31.4%; HR 1.17, 95% CI: 1.05–1.31, *p* = 0.005). However, the higher CD4 count group (*n* = 776) showed no significant difference from their controls (37.7% vs. 34.3%; HR 1.07, 95% CI: 0.94–1.21, *p* = 0.30). In contrast, the lower CD4 count group (*n* = 255) had a significantly higher risk (33.6% vs. 22.5%; HR 1.69, 95% CI: 1.34–2.14, *p* < 0.001).

**Conclusions:**

An HIV diagnosis was associated with increased job separation risk overall. However, using background-matched controls, the higher CD4 count group showed no increased risk. This suggests that HIV diagnosis itself may not increase the risk of job separation among individuals diagnosed at an early stage.

## Introduction

The widespread availability of antiretroviral therapy (ART) has dramatically improved the prognosis of people living with HIV (PLWH) [1–5]. With early diagnosis and sustained ART adherence, life expectancy for PLWH now approaches that of the general population [4, 5]. In this context, the Joint United Nations Programme on HIV and AIDS (UNAIDS) proposed a new target: ensure that 90% of people with viral load suppression have good health-related quality of life (HRQOL) [6]. As survival has improved, research focus has shifted toward HRQOL, including physical, psychological, and social well-being [7].

Employment is an essential factor in determining the HRQOL of PLWH because nearly 90% of HIV diagnoses are made in their working years [8]. Previous reports have found that employment enhances an individual’s self-esteem and dignity in PLWH [9]. Employment is also known to be associated with lower risk of mental health conditions such as depression [10, 11], improvement of adherence to the treatment [12], and enhanced quality of life [12].

Previous studies have investigated the employment status among PLWH. For example, a 2010 single-center cross-sectional study in the UK reported that approximately 74% of PLWH were engaged in some kind of work, and 51% in full-time paid employment [13]. Higher viral loads [14], frailty [15], psychological distress [16], a poor self-report of health [16], and self-reported experience of HIV-related discrimination at work [14] were associated with unemployment. In Japan, an online survey conducted in 2018 revealed that 10% of PLWH had experienced leaving their jobs due to their HIV status [17]. A 2013 study in Japan also revealed that job loss due to HIV status was experienced by 43.1% and 17.1% of unemployed and employed PLWH, respectively [18]. The reasons for job loss included poor health, followed by voluntary resignation and wrongful dismissal [18]. However, most existing studies on employment status among PLWH have been descriptive and lacked non-PLWH controls, making it difficult to assess the excess risk of job separation associated with HIV status.

To address this gap, we conducted a matched cohort study using a large-scale Japanese administrative claims database, comparing job separation risk between newly diagnosed PLWH and non-PLWH controls matched on key background characteristics.

## Methods

### Study design and data source

This study employed a retrospective matched cohort design. We used data from the JMDC claims database, which comprises records from 79 corporate health insurance societies in Japan, covering employees and their dependents [19]. In the Japanese healthcare system, employer-based health insurance covers approximately 60% of the population. The study period extended from January 2005 to August 2023. The JMDC claims database contained 17,656,319 enrollees during the study period. This population accounts for approximately 14.0% of the Japanese population, based on the 2020 national census [20]. The claims database includes diagnostic codes based on the International Classification of Diseases, Tenth Revision (ICD-10), prescription records, and details of medical procedures. It also contains demographic information for each participant, such as age, sex, presence of dependent family members, and dates of insurance enrollment and disenrollment.

### Definition of the newly diagnosed PLWH cohort

We included only male primary insured individuals. In Japan, the vast majority of people living with HIV are men, and the number of women with HIV in this database was too small for meaningful statistical analysis. In addition, employment patterns and job separation rates may differ between men and women. By requiring that the index date occurred during active insurance enrollment, we ensured that all participants were employed at the time of their HIV diagnosis.

HIV diagnosis was defined based on a confirmed HIV/AIDS diagnosis (ICD-10 codes: B20–B24) and receipt of antiretroviral therapy, according to the Anatomical Therapeutic Chemical (ATC) classification system (ATC code: J05C). A new HIV diagnosis was defined as the first record of an HIV-confirming diagnosis and initiation of antiretroviral therapy, accompanied by either an HIV screening or confirmatory test performed within 90 days before or after the diagnosis date. The date of the HIV-confirming diagnosis was defined as the index date. Participants who were younger than 18 or older than 59 years at the index date were excluded.

### Control group selection and matching

For each participant in the newly diagnosed PLWH group, we selected five participants who had no record of an HIV diagnosis or antiretroviral prescription. Controls were selected without replacement, meaning that a participant was selected as a control only once. Each control participant was assigned the same index date as their matched PLWH counterpart. To be eligible for selection as a control, the individual had to be enrolled in the insurance database on the index date. Exact matching was performed based on the following five criteria: age at the index date, the month and year of insurance enrollment, presence of dependent family members, history of psychiatric disorders (ICD-10 codes: F00-F99), and history of malignant tumors (ICD-10 codes: C00-C97). To avoid adjusting for potential mediators on the causal pathway from HIV diagnosis to job separation—such as psychiatric disorders or AIDS-related malignancies caused by the HIV diagnosis itself—only diagnoses recorded more than 90 days prior to the index date were considered for matching.

### Statistical analysis

The primary outcome was job separation, which was defined as disenrollment from insurance before the age of 60, not due to death, and occurring before August 31, 2023. Participants who remained enrolled beyond this date, reached the age of 60, or died during follow-up were considered censored. In this study, job separation refers to the termination of the employment relationship with the employer, which may occur due to voluntary resignation, job changes to another company, or involuntary dismissal.

We used the Kaplan-Meier method to estimate and plot survival curves, illustrating the cumulative incidence of job separation in the PLWH group and the control group. The log-rank test was conducted to compare the survival distributions between the two groups. To estimate the relative risk of job separation, we calculated hazard ratios (HRs) and 95% confidence intervals (CIs) using a stratified Cox proportional hazards model. The stratification was based on the matched sets.

All statistical analyses were performed using Python version 3.10.14 and Stata/MP 18.0. A p-value less than 0.05 was considered statistically significant.

### Subgroup analysis

Because laboratory test results including CD4 counts are not available in claims data, we conducted a subgroup analysis based on prescriptions for *Pneumocystis jirovecii* pneumonia (PJP) prophylaxis or treatment, which serve as a proxy for CD4 + T-cell count < 200 cells/µL [21]. PJP-related prescriptions were defined as receipt of atovaquone, sulfamethoxazole-trimethoprim, or inhaled pentamidine within 90 days before or after the index date. Those with and without such prescriptions were designated as the lower and higher CD4 count groups, respectively. Patient characteristics were compared between the two groups using the chi-square or Fisher’s exact test for categorical variables and the Mann-Whitney U test for continuous variables. The primary outcome was analyzed separately for each subgroup and their respective matched controls.

### Ethics approval

Informed consent to participate was waived by the Research Ethics Committee of the University of Tokyo because this study used anonymized secondary data (approval number: 2024216NIe). This study was conducted in accordance with the Declaration of Helsinki.

## Results

### Patient selection and matching

**Fig. 1:**
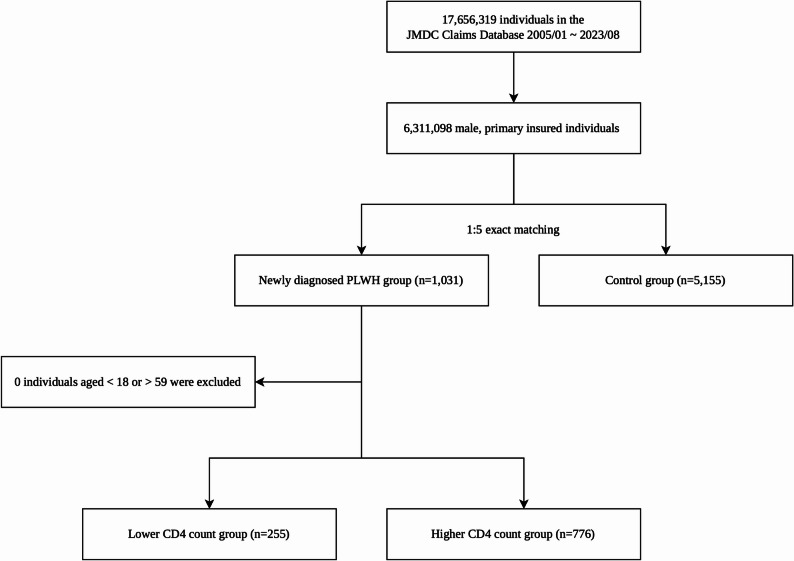
Flow diagram of participant selection. This figure illustrates the selection process for the study cohort from the JMDC claims database. From the database, male, primary insured individuals were selected. A total of 1,031 individuals with newly diagnosed HIV and 5,155 matched controls were included in the study. The PLWH group was further stratified into the lower CD4 count group (*n*=255) and the higher CD4 count group (*n*=776) based on PJP-related prescriptions. Abbreviations: PLWH, people living with HIV; PJP, Pneumocystis jirovecii pneumonia

Among 6,311,098 male enrollees who were the primary insured individuals identified in the dataset, all 1,031 newly diagnosed PLWH and their matched non-PLWH controls (*n* = 5,155, 1:5 matching) were included in this study (Fig. 1).

### Baseline characteristics

Baseline characteristics of the cohort are presented in Table 1. The median age at the index date was 36 years (interquartile range [IQR]: 30–44 years), and 11.2% (115/1,031) had dependent family members. A history of psychiatric disorders was present in 5.8% (60/1,031), and a history of malignant tumors was present in 0.5% (5/1,031) (Table 1).

**Table 1:** Characteristics of the participants at the index date

Characteristics	Newly diagnosed PLWH group				Control
	Total *n* = 1,031	Lower CD4 count *n* = 255	Higher CD4 count *n* = 776	*p* value ^a^	Total *n* = 5,155
Age ^b^ -year	36 (30–44)	41 (34–47)	35 (29–42)	< 0.001	36 (30–44)
18–29	242 (23.5%)	30 (11.8%)	212 (27.3%)		1210 (23.5%)
30–39	383 (37.1%)	76 (29.8%)	307 (39.6%)		1915 (37.1%)
40–49	302 (29.3%)	106 (41.6%)	196 (25.3%)		1510 (29.3%)
50–59	104 (10.1%)	43 (16.9%)	61 (7.9%)		520 (10.1%)
Dependent family members	115 (11.2%)	45 (17.6%)	70 (9.0%)	< 0.001	575 (11.2%)
Psychiatric disorders	60 (5.8%)	24 (9.4%)	36 (4.6%)	0.008	300 (5.8%)
Malignant tumors	5 (0.5%)	1 (0.4%)	4 (0.5%)	0.76	25 (0.5%)
Year of HIV diagnosis				0.46	
2005–2010	24 (2.3%)	1 (0.4%)	23 (3.0%)		
2011–2015	123 (11.9%)	40 (15.7%)	83 (10.7%)		
2016–2020	571 (55.4%)	138 (54.1%)	433 (55.8%)		
2021–2023	313 (30.4%)	76 (29.8%)	237 (30.5%)		

*Abbreviations*: *PLWH *People living with HIV

^a^p value for comparison between the lower and higher CD4 count groups

^b^median (interquartile range)

Among the PLWH group, 24.7% (255/1,031) received PJP-related prescriptions and were classified as the lower CD4 count group. Compared with the higher CD4 count group, participants in the lower CD4 count group were older (median age [IQR]: 41 [34–47] vs. 35 [29–42] years, *p* < 0.001), more likely to have dependent family members (17.6% [45/255] vs. 9.0% [70/776], *p* < 0.001), and more likely to have a history of psychiatric disorders (9.4% [24/255] vs. 4.6% [36/776], *p* = 0.008), while prevalence of a history of malignant tumors did not differ significantly (0.4% [1/255] vs. 0.5% [4/776], *p* = 0.76). Also, the year of HIV diagnosis did not differ significantly (median year of diagnosis [IQR]: 2019 [2017–2021] vs. 2019 [2017–2021], *p* = 0.46).

### Overall risk of job separation

**Fig. 2:**
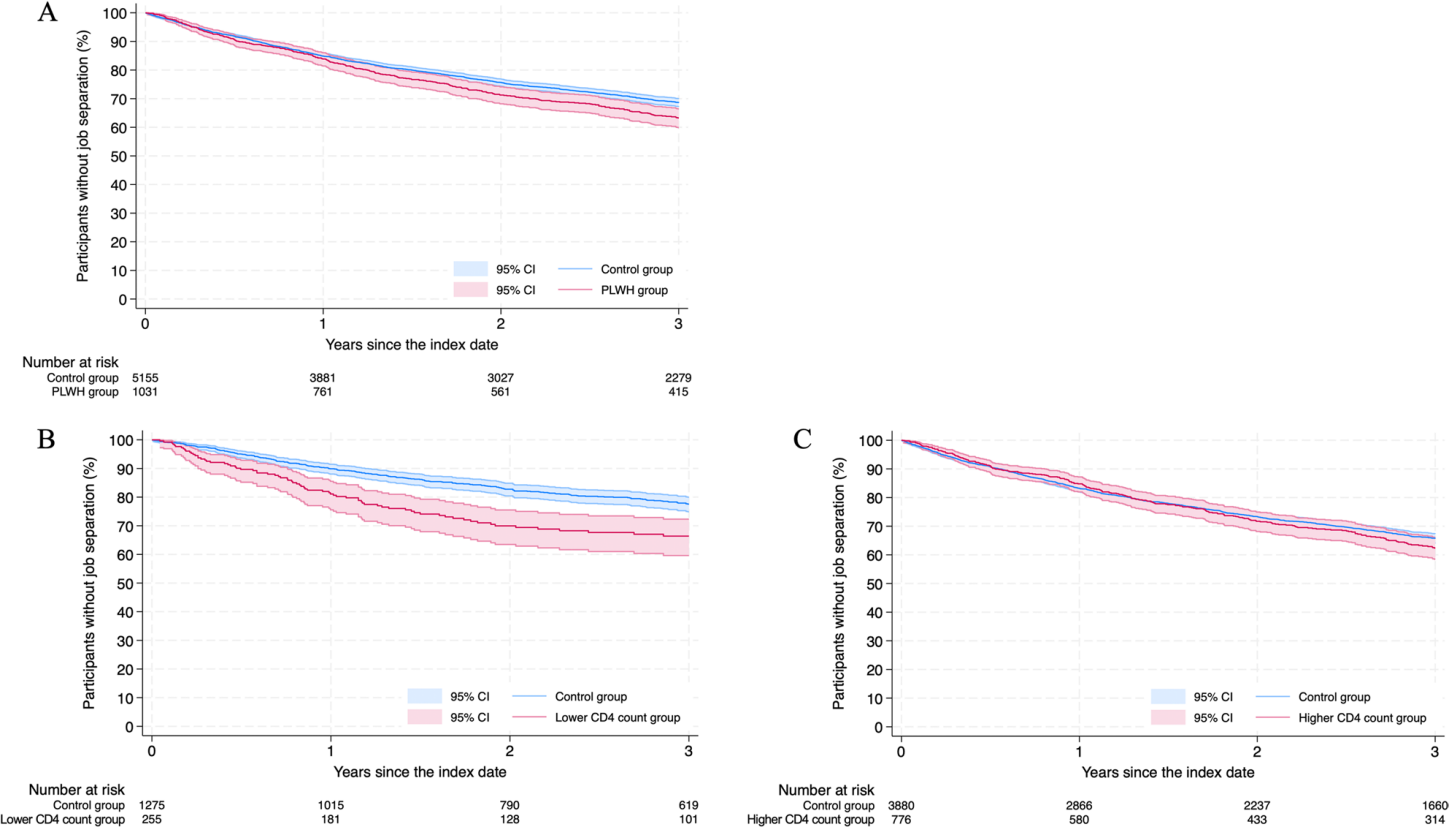
Kaplan-Meier Survival Curves for Job Separation. Kaplan-Meier survival curves for job separation over a three-year period between the PLWH group (red line) and the matched non-PLWH control group (blue line). The y-axis represents the participants without job separation as a percentage, while the x-axis shows the observation period in years. The shaded areas represent the 95% CI for each group. 2A. Overall comparison between the PLWH group and the control group 2B. The lower CD4 count group vs. their matched control group. 2C. The higher CD4 count group vs. their matched control group. Abbreviations: PLWH, people living with HIV; CI, confidence intervals

Within three years after the index date, the incidence of job separation was higher in the PLWH group, with a cumulative incidence of 36.8% (95% CI: 33.6–40.2) compared with 31.4% (95% CI: 30.0–32.8) in the control group (*p* = 0.004 by log-rank test). The HR for job separation was 1.17 (95% CI: 1.05–1.31, *p* = 0.005) (Fig. 2A).

### CD4-stratified risk of job separation

The lower CD4 count group had a significantly higher cumulative incidence of job separation (33.6%, 95% CI: 27.8–40.4) compared with their specific matched control group (22.5%, 95% CI: 20.0–25.1; *p* < 0.001), with the HR of 1.69 (95% CI: 1.34–2.14, *p* < 0.001) (Fig. 2B). The Kaplan-Meier curve for this group showed a steep initial decline during the early phase of follow-up, indicating that approximately two-thirds of the job separations occurred within the first year after the index date. Thereafter, the curve flattened, showing a trajectory similar to that of the control group. In contrast, the higher CD4 count group did not show a significant difference in the cumulative incidence of job separation compared with their matched controls (37.7%, 95% CI: 34.0–41.6 vs. 34.3%, 95% CI: 32.7–35.7; log-rank *p* = 0.27). The HR was 1.07 (95% CI: 0.94–1.21; *p* = 0.30) (Fig. 2C).

## Discussion

We retrospectively analyzed the impact of HIV diagnosis on job separation using a large health insurance claims database in Japan. To the best of our knowledge, this is the first quantitative study to evaluate the impact of HIV diagnosis on job separation, comparing with the matched non-PLWH cohort. Our analysis demonstrated that individuals diagnosed with HIV who had relatively preserved immune function (i.e., those classified as the higher CD4 count group) did not show an increased risk of job separation. In contrast, those with presumably advanced immunodeficiency at diagnosis (i.e., those classified as the lower CD4 count group) showed an increased risk of job separation compared with the matched non-PLWH, particularly within the early period following HIV diagnosis.

It is noteworthy that an HIV diagnosis did not increase the risk of job separation among individuals with higher CD4 counts. These findings suggest that if HIV is diagnosed before the onset of advanced immunodeficiency, the direct impact of the diagnosis itself may not significantly increase the risk of job separation, which may help alleviate employment-related concerns among PLWH.

Our findings contrast with previous literature that identifies social factors such as stigma, internalized stigma, and workplace discrimination as risk factors for unemployment among PLWH [18]. While this could suggest that HIV-related discrimination is less prevalent in Japan, a more careful interpretation is required. For instance, a questionnaire revealed that only about 18% of PLWH in Japan disclose their HIV status at work, which is lower than in other countries [17]. This low rate of disclosure may lower the risk of direct workplace discrimination, while opportunities for support can also be limited. The relationship between social stigma and job separation is complex, and further research is needed to elucidate these relationships in more diverse populations and employment settings.

The higher rate of early job separation among participants in the lower CD4 count group was a key observation. This trend is consistent with previous reports suggesting that most HIV-related job loss occurs within the first year of diagnosis [22]. These findings suggest that an HIV diagnosis may exert an acute impact on employment, particularly when occurring at an advanced stage. One primary driver is health-related complications caused by advanced immunodeficiency. This interpretation is supported by a single-center survey in Japan showing that health-related issues were the primary reason for job loss among PLWH [18]. A lower CD4 count [23, 24], an AIDS diagnosis, and a delayed diagnosis [25, 26] have been reported as significant predictors of job loss in PLWH. Beyond direct clinical symptoms, the necessity for intensive initial treatment may lead to hospitalization or prolonged medical leave during the early treatment period. Such structural challenges may in turn trigger disclosure-related workplace consequences, stigma, or inadequate employment protection structures, which could increase the risk of job separation. These findings highlight the importance of early HIV diagnosis and prompt initiation of care to support continued employment and overall well-being among PLWH.

In our study, participants in the lower CD4 count group tended to be older, more likely to have dependent family members, and were more likely to have a history of psychiatric disorders. The association between older age and receipt of PJP prophylaxis is particularly noteworthy, as it aligns with consistent findings from a Japanese study reporting that higher age was associated with lower CD4 cell count status (< 350 cells/µL) at diagnosis (adjusted odds ratio [aOR] 2.21, 95% CI 1.88–2.59, ≥ 45 vs. ≤29 years) [27]. This finding is also consistent with research from the United States and Tanzania, which identify older age as a strong predictor of a late HIV diagnosis [28, 29]. Besides the natural time required for disease progression, this trend is thought to be driven by a dual failure in risk perception: older individuals often do not consider themselves at risk of HIV, and healthcare providers may be less likely to recommend HIV testing to them. The finding that individuals with dependent family members were more common in the lower CD4 count group warrants careful interpretation, as research directly linking family status to late diagnosis in Japan is scarce. However, previous studies have established that heterosexual contact is a significant risk factor for late presentation [27]. Furthermore, among men who have sex with men (MSM), being married has been shown to be negatively associated with HIV testing [30]. It is plausible that our observation regarding dependent family members may be partially explained by these established risk factors, where family status could be intertwined with transmission routes and testing behaviors that lead to delayed diagnosis [31].

A key strength of our study is the use of background-matched controls to assess the impact of HIV diagnosis on job separation. Particularly, month and year of insurance enrollment were matched, which ensures that the employment histories before the index date were similar between PLWH and non-PLWH, and our comparisons were less likely to be affected by changes in the global economic situations, such as recession. The overall risk did not differ substantially between the lower CD4 count group and the higher CD4 count group, likely due to baseline differences as discussed above; those in the higher CD4 count group were generally younger, and younger individuals tend to have a higher risk of job separation [32]. By matching with non-PLWH controls, we were able to highlight two important findings: a higher early risk of job separation among participants in the lower CD4 count group, and no excess risk among those in the higher CD4 count group.

From a policy perspective, these findings highlight the importance of early HIV diagnosis and sustained clinical management. The absence of an increased risk of job separation among individuals with higher CD4 counts suggests that HIV infection itself may not be an inherent barrier to continued employment when treatment is initiated before the onset of advanced immunodeficiency. This message may be important for public health efforts to promote HIV testing, as early diagnosis not only improves clinical outcomes but may also help preserve employment and social stability among people living with HIV.

Our study has several limitations. First, the participants in this study may not fully reflect the general population of Japan. We used the JMDC claims database, which primarily consists of records of employees from larger enterprises. According to research by the Ministry of Health, Labour and Welfare, employees in large companies are less likely to change their jobs [32]. Previous studies have also shown that PLWH with full-time employment and higher social status have a lower risk of unemployment [15, 22]. Therefore, our findings may not be extrapolated to PLWH who worked for smaller companies at the time of their diagnosis. Because workers in smaller companies or precarious employment may face greater job insecurity and weaker employment protections, the impact of an HIV diagnosis on job separation may be greater than our estimates.

Second, insurance disenrollment may occur for several reasons, including job loss, job change, migration, or retirement, and these scenarios cannot be distinguished in the claims database. Retirement is unlikely to have substantially affected our results because individuals were censored at 60 years of age. Although we could not differentiate involuntary job loss from voluntary job changes, both situations may be influenced by HIV-related stigma or workplace difficulties following diagnosis. Therefore, the absence of an increased risk of job separation in the higher CD4 count group is unlikely to be substantially affected by this limitation.

Third, unmeasured confounders may exist. Differences in region, sexual orientation, education level, and occupational category could be confounding factors. Previous population-based surveys in Japan have suggested that gay and bisexual men report higher levels of workplace harassment and greater concerns about job loss compared with heterosexual men [33]. Such social vulnerabilities may influence employment stability and therefore represent potential unmeasured confounders in our analysis. These factors would be expected to bias the results toward a higher risk of job separation among PLWH rather than toward the null. Therefore, the absence of an increased risk of job separation in the higher CD4 count group suggests that our findings are unlikely to be explained solely by such confounding. In addition, the Kaplan–Meier curve for this group showed a steep initial decline during the first year followed by a trajectory parallel to that of the controls, suggesting that the observed early job separation is likely driven by the acute impact of advanced HIV disease rather than unmeasured socio-demographic confounders.

Finally, our study focused exclusively on men. In Japan, the vast majority of PLWH are male, and only 24 women met the eligibility criteria, precluding meaningful analysis. In addition, the social and occupational contexts surrounding HIV infection among women may differ from those among men, including differences in employment patterns and stigma. Therefore, our findings may not be generalizable to women living with HIV. Future studies with larger female HIV populations are needed to clarify these gender-specific impacts.

## Conclusions

In conclusion, we conducted a retrospective matched cohort study to assess the impact of HIV diagnosis on job separation among male workers in Japan. HIV diagnosis was associated with an increased risk of job separation overall. However, using background-matched controls, the higher CD4 count group showed no increased risk compared with their matched controls, whereas the lower CD4 count group had a significantly higher risk, particularly during the first year after diagnosis. These findings suggest that early HIV diagnosis, before the onset of advanced immunodeficiency, may not be associated with an increased risk of job separation.

## Data Availability

The claims data analyzed in this study are not publicly available as they were obtained from a commercial third-party database provider, JMDC Inc., and are subject to a data use agreement that prohibits redistribution. Researchers seeking to access this dataset can inquire directly with JMDC Inc. The statistical code used to generate the findings of this study can be made available by the corresponding author upon reasonable request.

## Abbreviations

ART: Antiretroviral therapy
ATC: Anatomical Therapeutic Chemical classification system
aOR: Adjusted odds ratio
CI: Confidence interval
HR: Hazard ratio
HRQOL: Health-related quality of life
ICD-10: International Classification of Diseases Tenth Revision
MSM: Men who have sex with men
PJP: Pneumocystis jirovecii pneumonia
PLWH: People living with HIV
UNAIDS: Joint United Nations Programme on HIV and AIDS
